# Sagittal jaw position in relation to body posture in adult humans – a rasterstereographic study

**DOI:** 10.1186/1471-2474-7-8

**Published:** 2006-01-31

**Authors:** Carsten Lippold, Gholamreza Danesh, Markus Schilgen, Burkhard Drerup, Lars Hackenberg

**Affiliations:** 1 Poliklinik für Kieferorthopädie, Universität Münster, Waldeyerstr. 30, 48149 Münster, Germany; 2 Akademie für Manuelle Medizin an der Universität Münster, Waldeyerstr. 30, 48149 Münster, Germany; 3 Klinik und Poliklinik für Technische Orthopädie und Rehabilitation, Universität Münster, Robert-Koch-Straße 30, 48149 Münster, Germany; 4 Poliklinik für Allgemeine Orthopädie, Universität Münster, Albert-Schweitzer-Straße 33, 48149 Münster, Germany

## Abstract

**Background:**

The correlations between the sagittal jaw position and the cranio – cervical inclination are described in literature. Only few studies focus on the sagittal jaw position and the body posture using valid and objective orthopaedic examination methods. The aim of this study was to test the hypothesis that patients with malocclusions reveal significant differences in body posture compared to those without (upper thoracic inclination, kyphotic angle, lordotic angle and lower lumbar inclination).

**Methods:**

Eighty-four healthy adult patients (with a mean age = 25.6 years and ranging from 16.1 to 55.8 years) were examined with informed consent. The orthodontic examination horizontal overjet (distance between upper and lower incisors) was determined by using an orthodontic digital sliding calliper. The subjects were subdivided in respect of the overjet with the following results: 18 revealed a normal overjet (Class I), 38 had an increased overjet (Class II) and 28 had an reversed overjet (Class III). Rasterstereography was used to carry out a three – dimensional back shape analysis. This method is based on photogrammetry. A three-dimensional shape was produced by analysing the distortion of parallel horizontal white light lines projected on the patient's back, followed by mathematical modelling. On the basis of the sagittal profile the upper thoracic inclination, the thoracic angle, the lordotic angle and the pelvic inclination were determined with a reported accuracy of 2.8° and the correlations to the sagittal jaw position were calculated by means of ANOVA, Scheffé and Kruskal-Wallis procedures.

**Results:**

Between the different overjet groups, no statistically significant differences or correlations regarding the analysed back shape parameters could be obtained. However, comparing males and females there were statistically significant differences in view of the parameters 'lordotic angle' and 'pelvic inclination'.

**Conclusion:**

No correlations between overjet and variables of the thoracic, lordotic or the pelvic inclination could be observed.

## Background

The cervical column is in close functional and morphological relationship to structures of the masticatory system [1,2]. It is typical that patients revealing a short face morphology show a backward inclination and patients with long face morphology are characterised by a forward inclination of the cervical column [3,4]. These findings are recognized by many orthodontic practitioners and the documentation often remains semi – objective and anecdotal. A number of studies used lateral cephalometric radiographs of the head to analyse the posture of the head and neck region (cranio-cervical angles; cervical inclination to horizontal or vertical reference planes) [1,3,5-9]. Solow et al. analysed 120 Danish male dental students (age 20–30) and revealed low significant correlations between head inclination and craniofacial morphology [4,5]. All the cephalometric radiographs in these studies were made in a self-balanced mirror position. Festa et al. [6] used lateral cephalographs to evaluate the relationship between mandibular length and cervical inclination and could find positive correlations between these parameters in 70 Caucasian women (mean age 27.4 years) with skeletal class II malocclusion. D'Attilio et al. [7] also revealed a correlation between cervical lordosis and mandibular position, mandibular length, mandibular divergency and overjet. Conversely, some studies dismiss the existence of specific orthopedic findings in patients with different sagittal jaw positions [10-12]. Concerning the close functional correlations of the cervical and thoracic spine, the back shape of the thoracic and pelvic region seems to be of interest regarding relationships between sagittal jaw position and body posture [12]. Michelotti et al. [2] stated in a review article that there exists some evidence for correlations between jaw position and cervical inclination, however for the lower vertebrae these correlations tend to disappear.

Based on the results of the cited studies we set up the working hypothesis at the beginning of this prospective study that statistical significant correlations between different sagittal jaw positions and the back shape profile (kyphotic and the lordotic angle) exist. Aim of the presented study was to evaluate statistical significant relationships between the upper thoracic inclination, kyphotic angle, lordotic angle and pelvic inclination in healthy adult patients with different occlusal parameters using rasterstereographic back shape analysis and orthodontic examination.

## Methods

### Subjects

The sample of investigated patients comprised 84 healthy adults (54 women, 30 men; mean age = 25.6, range 16.1 – 55.4 years; mean body weight = 69.6; mean height 174.3 cm). 66 subjects were admitted to the Department of Orthodontics, University of Münster for combined orthodontic-orthognathic surgery treatment. The control group was composed of 18 dental students from the University of Münster.

Of the patients 38 subjects revealed an increased overjet (27 women, mean age = 26.58, SD 6.92; 11 men, mean age = 25.93, SD 6.92), 28 subjects an reversed overjet (13 women, mean age = 23.11, SD 7.23; 15 men, mean age = 24.59, SD 9.55) and the control group of 18 dental students a normal overjet (14 women, mean age = 25.38, SD 6.21; 4 men, mean age = 28.32, SD 5.42).

No patients in the study sample showed a history of motor or neurological problems, and there were no subjects who had experienced orthopaedic trauma or had any other diagnosed health problems. The subjects gave their informed consent to the experimental procedure according to the Helsinki criteria and the local Ethics Committee.

### Equipment

#### a) Orthodontic examination

Of all subjects alginate impressions of the maxillary and mandibular arch were taken in order to fabricate dental casts oriented in a three – dimensional direction by means of a wax – bite plate. The overjet (sagittal distance between upper and lower incisors) [14] was measured using an electronic digital sliding calliper (JOCAL, C.E. Johansson, Eskilstuna, Sweden). The subjects were subdivided into three different groups according to the determined overjet: Class I "normal overjet" 1 – 3 mm, Class II "increased overjet" 4 – 10 mm and Class III "reversed overjet" < 1 mm. Patients admitted for combined orthodontic- and orthognathic surgery treatment revealed increased or reversed overjet (Class II and III). Class I was composed of 18 dental students. This classification was carried out according to standard orthodontic classification of sagittal occlusion parameters [14]. For supplementary differentiation of the overjet the distances were noted in 0.5 mm steps.

#### b) Rasterstereographic back shape analysis

Rasterstereography [15,16] (Formetric 2, Diers International GmbH, Schlangenbad, Germany) may be classified as a method of 3-D optical surface measurement based on the principles of photogrammetry.

It uses a system of horizontal parallel white light lines projected onto the back surface of the patient. Observing this light raster from a direction that is different from the projection reveals shape information from the distortion of the white lines [17]. The synchronous projection and registration of all light lines in one instant reduces the measurement time of all the surfaces to typically 0.25 sec., therefore this method is the proper choice in back shape measurement. Due to the high accuracy of the measurement of the coordinate data (typically 0.1 mm), rasterstereography allows the application of sophisticated method and the automatic localization of anatomical landmarks on the back surface, like for example, the spinous processus of C7 vertebra (vertebra prominens) or the right and left dimple points in the pelvic region (spinae iliacae posterior superior). On the basis of these landmarks the sagittal back profile is established automatically and a set of shape parameters characterizing the back profile is provided [18].

**Figure 1:**
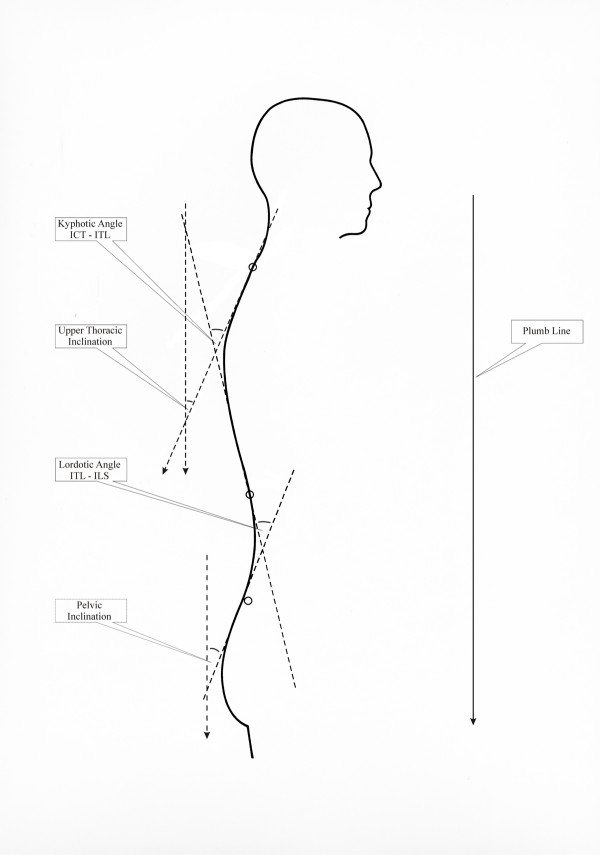
**Lateral Profile Analysis derived from Rasterestereographic data. **Outline of the sagittal profile. Three inflectional points along the profile are marked by open circles – the cervicothoracic (ICT), the thoracolumbar (ITL) and the lumbo-sacral inflection point (ILS). The tangents to the inflectional points span two characteristic angles of the profile – the kyphotic angle (KA) is spanned by the tangent lines in ICT and ITL; in an analogous way the lordotic angle (LA) is spanned by the tangents in ITL and ILS. Two other angles provide orientation data with respect to the vertical line (plumb line); 1) the angle of the upper thoracic inclination (UTI), which is virtually the angle spanned by the vertical and the ICT tangent, 2) the angle of the pelvic inclination (PI), virtually the angle spanned by the vertical and the tangent ILS

Figure 1 shows the outline of a sagittal profile. Along the profile three inflectional points are indicated by open circles – the cervico-thoracic (ICT), the thoraco-lumbar (ITL) and the lumbo-sacral inflection point (ILS). The tangents to the inflectional points span two characteristic angles of the profile – The kyphotic angle (KA) is spanned by the tangent lines in ICT and ITL; in an analogous way the lordotic angle (LA) is spanned by the tangents in ITL and ILS. According to their definition concerning the inflectional tangents, these angles provide maximum angles which are to be measured along the profile. The reproducibility of a single rasterstereographic measurement of the kyphosis and lordosis angle is specified to 2.8° [19]. Accordingly, the reproducibility of the inclination measurements in respect of the vertical line is expected to 2°.

The kyphotic and lordotic angles are relative angles without reference to the vertical line. Two other angles provide orientation data with respect to the vertical line (plumb line); 1) the angle of the upper thoracic inclination (UTI), which is virtually the angle spanned by the vertical and the ICT tangent, 2) the angle of the pelvic inclination (PI), virtually the angle spanned by the vertical and the tangent ILS (Figure 1).

For each patient a rasterstereographic recording is taken in a standardized position and posture – the patient stands barefoot in relaxed posture. The positioning with respect to the measurement system was carried out according to the recommendations of the supplier.

#### c) Data analysis

SPSS 12.0 (Lead Tech., Chicago, IL, USA) software was employed to perform the data analysis. The posture parameters involved in the measurements were: UTI, KA, LA and PI. in order to determine the craniofacial morphology, the Angle Classification and the overjet were considered. ANOVA, Scheffé and Kruskal-Wallis procedures were used to test our hypothesis. Level of significance was set at p < 0.05.

## Results

The orthodontic examination of the subjects revealed a mean overjet of -2.11 mm (standard deviation 3.72), varying between -10 and 6.0 mm. 18 (21.4%) of the subjects belong, according to their horizontal overjet, to Class I, 38 (45.2) to Class II and 28 (33.3%) to Class III. The measurement data (mean values and SD) for the kyphotic and lordotic angle, the upper thoracic and the pelvic inclination are presented in additional file 1. The statistical analysis (ANOVA) of the back profiles in the patients grouped according to their sagittal jaw position does not show any significant differences, neither for the upper thoracic inclination (p > 0.05), the kyphotic angle (p > 0.05), the lordotic angle (p > 0.05) nor for the pelvic inclination (p > 0.05). Likewise, further metric differentiation of the overjet in steps of 0.5 mm showed no significant differences (p > 0.05). No significant correlation between the overjet and the upper thoracic inclination (R2 = 0.022), the kyphotic angle (R2 = 0.011), the lordotic angle (R2 = 0.012) and the pelvic inclination (R2 = 0.02) could be found.

However, the statistical significant differences (Kruskal-Wallis) between the sexes determined in view of the lordotic angle (p < 0.01) and the pelvic inclination (p < 0.01) were revealed. Even with an additional subdivision of the groups (Class I, II and III) confirmed these differences (p < 0.01).

## Discussion

The initial hypothesis of this prospective study stated that patients with different sagittal jaw positions show different back shape curvatures regarding the upper thoracic and the pelvic inclination to a vertical plumb line, the kyphotic angle and the lordotic angle. To test this hypothesis valid information using lateral cephalographs for the analysis of sagittal jaw position and the neutral vertebrae in the radiological Cobb-angle definition might have been used. However, arguments on possible radiation hazards would not justify this attempt. Therefore rasterstereography was established, allowing an examination of the back profile without radiation exposure but providing comparable accuracy and objectivity. It has been shown, that in the sagittal plane exists a reliable correlation between the spinal curvature and the back profile curvature [19]. The accuracy of the system was tested by Hackenberg et al. [20,21] on patients with idiopathic scoliosis after anterior and posterior correction and fusion and showed a good accuracy compared with the anterior-posterior thoracic radiographs. In order to simplify the examination technique Drerup et al. provided four parameters of the sagittal back shape profile to determine back shape in a standardized wise fashion [22].

In literature documentation about significant correlations between the jaw position and the parameters of the body posture often anecdotal and important questions are left unanswered [1,2]. The positive correlation between different skeletal facial patterns and the cervical inclination was analysed in a series of studies by Solow et al. and Huggare et al. [3,5,8,9]. Nobili et al. [12] used a "balance platform" (dynamometric platform for postural oscillation measurement) and revealed positive correlations between the sagittal jaw position and the body posture: the class II malocclusion was correlated to the anterior body posture and the class III malocclusion to the posterior oriented posture. Korbmacher et al. [1] concluded that studies of correlations between orthodontic and orthopedic findings often lack precise orthopedic measurement methods. Furthermore it was a demand of the same authors to provide prospective clinical trials in close cooperation with orthodontics and orthopaedics. The presented study fulfills this demand because an interdisciplinary team (orthodontists, maxillofacial surgeons, orthopaedic surgeons and biomechanic physicians) analysed the patients in this study. Even more sophisticated methods for orthopedical examination were used.

The results of the present study do not show any significant relationships between different sagittal jaw positions and kyphotic or lordotic angle, upper thoracic and pelvic inclination (p > 0.05). These results are in agreement with a critical review of the literature by Michelotti et al. [2], who postulated that there is some evidence between sagittal jaw position and cervical posture but not to body posture Nevertheless, the pelvic inclination and the lordotic angle show significant differences (p < 0.01) between the examined males and females. This is in agreement with Vialle et al. [23], Korovessis et al. [24,25], Legaye et al. [26], and Gelb et al. [27] but contrary to the results of Jackson et al. [28-30].

## Conclusion

The measurement of the body posture by means of rasterstereography is known to provide accurate information on the back shape and the sagittal profile without entailing radiographic strain for the patient. With this system no correlations between the sagittal jaw position and variables of the kyphotic, the lordotic or the pelvic inclination could be found. Further studies are necessary to provide cephalometric measurements of the craniofacial skeleton and to correlate them to the examined parameters used in the present study.

## Competing interests

The author(s) declare, that they have no competing interests.

## Authors' contributions

CL carried out the study design and initiated the study, participated in the data collection and drafted the manuscript. GD carried out the orthodontic analysis, datacollection, data processing and the statistical analysis. MS participated in the idea for this study, data collection and rasterstereographic analysis. BD participated in the design of the study and did the programming of the raterstereographic units that were used in this study. Moreover he helped to draft the manuscript. LH participated in its design and coordination and helped to draft the manuscript. All authors read and approved the final manuscript.

## Pre-publication history

The pre-publication history for this paper can be accessed here:



## Supplementary Material

Additional File 1 **Descriptive Statistics and ANOVA results of the Rasterstereographic Back Shape Analysis**. Descriptive Statistics (mean and SD) of Upper Thoracic Inclination (UTI), Kyphotic Angle (KA), Lordotic Angle (LA) and Pelvic Inclination (LA) for males, females and the total number of patients in Class I (normal overjet), Class II (enlarged overjet) and Class III (reversed overjet); p-values of the ANOVA results (* = statistically significant) among males and females. Missing values: KA (12 measurements) and LA (2 measurements) due to measurement problems. Click here for file
