# Supplementary material for: Sagittal jaw position in relation to body posture in adult humans – a rasterstereographic study
Source: BMC Musculoskelet Disord. 2006 Jan 31;7:8. doi: 10.1186/1471-2474-7-8 (PMC1379641; doi:10.1186/1471-2474-7-8)
Supplement: Additional File 1 — Descriptive Statistics and ANOVA results of the Rasterstereographic Back Shape Analysis. Descriptive Statistics (mean and SD) of Upper Thoracic Inclination (UTI), Kyphotic Angle (KA), Lordotic Angle (LA) and Pelvic Inclination (LA) for males, females and the total number of patients in Class I (normal overjet), Class II (enlarged overjet) and Class III (reversed overjet); p-values of the ANOVA results (* = statistically significant) among males and females. Missing values: KA (12 measurements) and LA (2 measurements) due to measurement problems. [file 1471-2474-7-8-S1.doc]

**Additional file 1.**

|  | **Class I mean ± SD [degree]** | | | | | **Class II**  **mean ± SD [degree]** | | | | | **Class III**  **mean ± SD [degree]** | | | | |
| --- | --- | --- | --- | --- | --- | --- | --- | --- | --- | --- | --- | --- | --- | --- | --- |
|  | **n** | **UTI** | **KA** | **LA** | **PI** | **n** | **UTI** | **KA** | **LA** | **PI** | **n** | **UTI** | **KA** | **LA** | **PI** |
| **male** | 4 | 33.8 ±7.1 | 48.4 ±10.5 | 34.9 ±4.1 | 20.5 ±1.3 | 11 | 34,8 ±4,9 | 53,9 ±7,6 | 37,6 ±9,3 | 21,9 ±8,2 | 15 | 36,4 ±4,7 | 50,7 ±6,7 | 34,3 ±10,8 | 20,1 ±7,9 |
| **female** | 14 | 36.9 ±4.9 | 52.7 ±7.8 | 43.6 ±8.5 | 28,6 ±5,3 | 27 | 34,2 ±6,4 | 48,2 ±7,1 | 46,0 ±7,2 | 29,7 ±5,9 | 13 | 34,6 ±6,9 | 51,0 ±7,9 | 46,1 ±6,5 | 28,4 ±4,9 |
| **total** | 18 | 36,3 ±5,4 | 51,8 ±8,4 | 41,5 ±8,5 | 26,8 ±5,9 | 38 | 34,4 ±6,0 | 50,2 ±7,6 | 43,7 ±8,6 | 27,4 ±7,4 | 28 | 35,6 ±5,8 | 50,8 ±7,0 | 39,8 ±10,8 | 23,9 ±7,8 |
| **p-value** |  | 0.33 | 0.37 | 0.07 | 0.009^*^ |  | 0.77 | 0.06 | 0.006^*^ | 0.002^*^ |  | 0.414 | 0.908 | 0.002^*^ | 0.003^*^ |
